# Syndrome-Based Prescription to Optimize Psychotropics: Are CHROME Criteria a Game Changer?

**DOI:** 10.3389/fpsyt.2021.662228

**Published:** 2021-04-22

**Authors:** Ruben Muñiz, Jorge López-Alvarez, Luis Agüera-Ortiz, Luis Perea, Javier Olazarán

**Affiliations:** ^1^Maria Wolff Foundation, Madrid, Spain; ^2^Servicio de Psiquiatría, Instituto de Investigación i+12, Hospital Universitario 12 de Octubre, Madrid, Spain; ^3^CIBERSAM, Madrid, Spain; ^4^Albertia Servicios Sociosanitarios, Madrid, Spain; ^5^Memory Disorders Clinic, HM Hospitals, Madrid, Spain; ^6^Neurology Service, University Hospital Gregorio Marañón, Madrid, Spain

**Keywords:** chemical restraint, dementia, neuropsychiatric symptoms, pharmacological treatment, quality prescription, psychotropic medications, neuropsychiatric syndrome

## Abstract

A variety of medical and social factors have contributed over the last decades to the overuse of psychotropic drugs in people with dementia. One social factor is probably the frequent failure to provide adequate person-centered care, be it in the community or in institutional settings. This unfortunate reality has been reacted upon with numerous guidelines to reduce prescriptions of the most dangerous drugs (e.g., neuroleptics). Each psychotropic drug prescription can in principle be assessed around three dimensions: (a) adequate, (b) inadequate, and (c) chemical restraint. The **CH**emical **R**estraints av**O**idance **ME**thodology (CHROME) defined chemical restraint as any prescription based on organizational convenience, rather than justified with medical diagnosis. Two validation studies revealed that one of the main medical reasons of over- and miss-prescriptions was symptom-based prescription. By switching to syndrome-based prescription, a large proportion of drugs could be de-prescribed and some re-adjusted or kept. Paucity of research and weakness of data are not conclusive about the adequacy of specific drugs for the myriad of cases presented by patients with dementia and comorbid conditions. Clinical practice, however, leads us to believe that even under optimal care conditions, psychotropics might still contribute to quality of life if based on an adequate diagnosis. This article explains the rationale that underlies a syndromic approach aimed at optimizing psychotropic treatment in people with dementia whose significant suffering derives from their thought, affective, or behavioral problems. The results of previous validation studies of this new methodology will be discussed and conclusions for future results will be drawn.

## Introduction

Polypharmacy is a major issue for the geriatric patient in general (1, 2). Among this group of patients, additional concern has been raised in elders with dementia regarding psychotropics, especially long half-life benzodiazepines and all type neuroleptics (3).

Evidence that inadequate prescriptions exposed patients to disproportionate risks of cognitive deterioration, vascular events, injurious falls, pneumonia, and death set off several complementary lines of initiatives. While many scholars focused on the most urgent problem, i.e., deprescribing (4–6), the concept of “chemical restraint” was elaborated in normative attempts to reduce risks associated with mostly wrongful prescriptions (e.g., Centers for Medicare and Medicaid Services, Interpretive Guidelines §483.13a; Gobierno de Navarra, Decreto Foral 221/2011; Deutsches Strafgesetzbuch (StGB) §239 Freiheitsberaubung).

In that context, prescribing optimization was neither priority, nor were there guidelines available to handle psychotropic prescribing of people with dementia in an all-encompassing way. An exception were the International Psychogeriatric Association (IPA) guidelines for the treatment of behavioral and psychological symptoms of dementia (BPSD) (7). These guidelines, which clearly recommend non-pharmacological treatment as first option, are however difficult to implement in institutions with overburdened staff. The scarcity of resources for adequate non-pharmacological and environmental interventions exerts pressure on physicians to compensate those shortcomings with medication. In addition, the BPSD approach may lead in practice to symptom-oriented prescriptions, which besides, has yet not proven satisfactory. Moreover, symptom-based prescribing could end up in psychotropic polypharmacy, since several BPSD may concur in the same person.

The paucity of research and the weakness of data derived from clinical trials about the efficacy of psychotropics in people with dementia can shed doubts on their usefulness (8). In everyday clinical practice though, neuropsychiatric symptoms appear even in persons where almost optimal care seems provided. When adequately diagnosed and carefully prescribed, the improvement of these cases provides an empirical base for the value of a psychopharmacological approach.

In this article we will describe the rationale for the development of a global syndromic approach that can help to optimize psychotropic treatment in people with dementia who display thought, affective, or behavioral problems. The results of previous validation studies of this new methodology will be discussed, and conclusions will be drawn for future research.

## Symptom, Syndrome, And Disease

Modern medicine received a definite boost with the advent of the clinical method, which traces an empirical link from symptoms, through syndromes, to the cause of disease. The scientific path based on observation and testing, which continues to this day, has contributed to the development of diagnostic methods and treatments for a multitude of processes and has been decisive for the well-being of our societies (9).

A medical process can be studied from three pathological perspectives: symptomatic, syndromic, and etiologic. Symptom refers to an observable behavior or state for which there is no implication that an underlying problem or physical etiology necessarily exists. It is the simplest level of analyzing a patient's manifestation. The next higher level of analysis is the syndrome, which is applied to a constellation of symptoms that occur together or co-vary over time. This concept carries some implications in terms of underlying pathology. The highest level of conceptual understanding is disease, which implies specific pathogenic processes and etiological factors (10). While Neurology has achieved important pathophysiological and etiological advances in the study of processes associated with cognitive impairment (9), Psychiatry has mainly remained in the syndromic field (11).

In contrast with neurodegenerative disorders, there is a lack of reliable biomarkers for psychiatric clinical pictures, possibly reflecting much more complex etiopathogenesis of disease, including not only genetic, but also psychological and social factors. In fact, internationally accepted diagnostic criteria, i.e., International Classification of Diseases (ICD) (12) and Diagnostic and Statistical Manual of Mental Disorders (DSM) (11), are basically expert consensus, based on syndromic analyses, rather than etiology. The role of environmental stress and psychological reaction may be determinant in a substantial proportion of psychopathological conditions at any age, which is consistent with the evanescent and self-limited nature of neuropsychiatric symptoms in dementia, even if not pharmacologically treated (13).

## Moving From Symptoms To Syndrome: The Chrome Approach

Despite being the most changeable and least reliable, the symptomatic approach is usually the most utilized in the description and treatment of non-cognitive symptoms of people with dementia (14–18). This approach may be useful to establish a classification and taxonomy of patient problems, as well as for the achievement of relief through non-pharmacological measures (e.g., environmental modification and psychological support), but may not be adequate for the pharmacological treatment (Table 1) (19, 20). In fact, when merely based on individual symptoms, drug treatment usually leads to over-prescription, unpredictable results, and undesirable side effects.

**Table 1:** Differences between symptom (BPSD) and syndrome approach (CHROME) to the neuropsychiatric manifestations of dementia.

	**BPSD^a^**	**CHROME**
Definition	Symptoms of disturbed perception, thought content, mood, or behavior that frequently occur in patients with dementia	Constellations of symptoms presenting specific core features and producing significant suffering or risk
Level of analysis	Description of signs and symptoms, based on patient observation (behavioral symptoms) or interview (psychological symptoms)	Signs and symptoms integration according to pre-defined patterns; identification of primary syndrome is encouraged
Taxonomy	Physical aggression, screaming, restlessness, agitation, wandering, culturally inappropriate behaviors, sexual disinhibition, hoarding, cursing, shadowing (behavioral symptoms) Anxiety, depressive mood, hallucinations, delusions (psychological symptoms)	Depression, anxiety, psychotic syndrome, impulsive syndrome, maniform syndrome, sleep disturbance
Biological substrate	Not necessarily	Distinct pathological substrate
Treatment	Primarily non-pharmacological	Initially non-pharmacological, pharmacological treatment is warranted for severe or refractory cases

a *Adapted from (18). BPSD: Behavioral and psychological symptoms of dementia; CHROME: chemical restraint avoidance methodology*.

With the aim of achieving a more rational and adequate pharmacological treatment, a group of experts defined six syndromes as core component of a multicomponent intervention to optimize psychotropic medication and, secondarily, eliminate chemical restraints in people with dementia (i.e., CHROME criteria) (21). A uniform adaptation of diagnostic criteria for some already defined syndromes was carried out (i.e., depression, anxiety, psychotic syndrome, sleep disturbance), a previous syndrome (impulsive syndrome) was slightly restricted, and a new one (maniform syndrome) was added. The existence of some common psychiatric “ways of falling ill,” sharing common neurobiological pathways, was implicitly assumed, independent of etiology and cognitive status (20, 22, 23). In addition, it was hypothesized that medications should be more effective the more the syndromes resembled those described in psychiatric patients without dementia, for whom the drugs were tested and approved.

The language of the DSM was utilized, as it is the most accepted and used by the scientific community (11). In fact, the DSM methodology fits perfectly with CHROME approach, as it is a fundamentally syndromic classification. Furthermore, the DSM leaves ample room for clinical judgment, which is critical in the neuropsychiatric diagnosis of patients with dementia, in whom, distinguishing between organic and reactive components is crucial (13). The authors believe that this syndromic approach can be helpful for non-psychiatrist physicians, particularly nursing home and family medicine physicians, who have less training in psychopathology, making it easier for those doctors to identify the problems to be treated.

The clinical characteristics of CHROME neuropsychiatric syndromes and the indicated medications are presented in Table 2, along with level of evidence of clinical effect, based on existing reviews (24–34). Additional background and discussion will be provided in the following sections.

**Table 2:** CHROME syndromes and indicated medications.

	**Core symptoms^a^**	**Duration**	**Indicated medications**	**Grade of evidence^b^ and supporting references**
Depression	Sadness, anhedonia, lack of hope	Most of the time for the last 2 weeks	SSRI, SNRI, other antidepressants (mirtazapine, vortioxetine, bupropion)	Low (24–27)
Anxiety	Excessive/unjustified fear, feeling of loss of control, somatic complaints, repetitive thoughts, or behaviors	Most of the time for the last 2 weeks	- SSRI, SNRI, other antidepressants (mirtazapine, trazodone) - Short/middle half-life BZD, gabapentin, pregabalin^c^ - Atypical antipsychotics^d^	Low (24, 28) Very low (28) Very low (28)
Psychotic syndrome	False beliefs or stories (ideas of theft, abandonment, prejudice, infidelity, etc.) or false perceptions (visual, auditory, etc.)	Most days for the last 7 days	Atypical antipsychotics	Moderate (24, 27, 29–31)
Impulsive syndrome	Lack of foresight or social tact	Most days for the last 2 weeks	- Serotoninergic medications (sertraline, citalopram, escitalopram, trazodone) - Antiepileptic drugs (valproate, gabapentin, pregabalin, carbamazepine, oxcarbamazepine, zonisamide), atypical antipsychotics^c^	Low (24, 27, 30, 32) Low (carbamazepine), very low (other antiepileptics), or moderate (antipsychotics) (24, 27, 29–31)
Maniform syndrome	Elevated mood, overestimation of own capabilities, feeling abnormally energetic, hyperactive, decreased need for rest	Most of the time for the last week	- Antiepileptic drugs (valproate, carbamazepine, oxcarbamazepine, topiramate), atypical antipsychotics (e.g., quetiapine) - Lithium^c^	Low (24) Very low (24)
Sleep disturbance	Loss of the physiological sleep-wake cycle (hypersomnia, insomnia, cycle inversion, fragmented sleep, etc.)	Most days for the last 2 weeks	- Short half-life benzodiazepines lorazepam, lormetazepam), benzodiazepine analogs (zolpidem, zopiclone), other medications (clomethiazole, trazodone, mirtazapine, gabapentin, pregabalin, melatonin), natural products (valeriana, passiflora) - Atypical antipsychotics (quetiapine, olanzapine)^c^	Low (mirtazapine, trazodone) or very low (rest) (24, 33) Very low (24)

a *To qualify for diagnosis, symptoms should produce significant distress, loss of functioning, or risk; in addition, symptoms should not be a mere consequence of cognitive deterioration, medical process, unmet basic needs, inadequate environment, or other neuropsychiatric symptom*;

b *according to GRADE system (34)*;

c *second choice*;

d *last choice. BZD, benzodiazepines; SNRI, serotonin and norepinephrine reuptake inhibitors; SSRI, selective serotonin reuptake inhibitors*.

## Depression

Depression is frequently reported in people with dementia. Traditionally associated with Alzheimer's and vascular dementia, a recent meta-analysis demonstrated that depression was also more frequent in dementia with Lewy bodies (DLB) and vascular dementia, compared to healthy subjects (35). In patients with dementia, depression was traditionally attributed to neuronal loss in brainstem monoaminergic nuclei (36, 37), later not confirmed though in ulterior studies (38).

Specific criteria for diagnosing depression in Alzheimer's disease were developed by an expert panel convened by the US National Institute of Mental Health (NIMH-dAD). Those criteria were derived from DSM-IV criteria for major depression (10), with a few key distinctions “to better reflect the clinical features of depression in Alzheimer's disease” (39). Briefly, the number, duration, and frequency of symptoms required for a diagnosis of depression were reduced, cognitive symptoms were eliminated, and social isolation/withdrawal and irritability were added as new symptoms (40). This approach, which yielded greater sensitivity than the DSM-IV (41), was considered adequate for the diagnosis of neuropsychiatric syndromes in patients with dementia and inspired definitions of the CHROME syndromes (Table 2).

In view of previous neuroanatomical studies (36, 37) and results obtained in non-demented patients, pharmacological treatment enhancing serotoninergic, noradrenergic, and dopaminergic transmission is indicated in patients with dementia and severe depression, provided response and safety is monitored, although significant effect was not deffinitely demonstrated in randomized controlled trials (24–27), which might be due to the exclusion of severely depressed patients (42).

## Anxiety

In dementia, anxiety, expressed as fear, restlessness, or somatic complaints, typically accompanies depression (19, 20, 43–45). However, since differences in terms of pharmacological and non-pharmacological treatment exist, a distinction of both syndromes was considered opportune (Table 2).

The high prevalence of anxiety in people with dementia, together with the widespread use of drugs for its relief, contrasts with the absence of scientific studies to support that practice (24, 27). Moreover, current standards of treatment of geriatric anxiety involve more of non-pharmacological approaches, which are the first line of recommended treatment. In severe cases, selective serotonin reuptake inhibitors (SSRI) represent the preferred long-term pharmacological treatment, while benzodiazepines should be restricted to short-term use, and quickly tapered, owing to multiple risks in the elderly, including confusion, agitation, falls, and drug dependence (28).

## Psychotic Syndrome

As it happens to other psychiatric symptoms, delusions and hallucinations usually appear together in dementia (19, 20, 43–45), thus warranting characterize a psychotic syndrome. However, unlike schizophrenic patients, delusions, and hallucinations show a low persistence in people with dementia (46). The postulated neural mechanisms include dopamine D_3_, serotonin, and cholinergic muscarinic receptors, among other chemical agents (47).

Previous diagnostic criteria were modified to be applicable to all types of dementia and to allow diagnosis after less duration of symptoms (48), also in consonance with criteria recently proposed by an expert panel of the International Psychogeriatric Association (49).

Randomized trials (24, 27, 29–31) and clinical experience support the use of neuroleptic treatment in patients with dementia who develop psychosis. However, given the high risks associated with long-term use of these medications (3, 50), antipsychotic discontinuation should be regularly assessed, even in case of good tolerance and response. Long-term antipsychotic use might be beneficial in a small proportion of patients, who suffered severe psychosis and showed good treatment response (31).

## Impulsive Syndrome

Impulsive syndrome, defined as a lack of foresight or social tact, includes uninhibited and aggressive behaviors, which often appear together in people with dementia (19, 45). For definition purposes, the term agitation was intentionally avoided due to widespread but unspecific utilization in clinical practice. Agitation may be used, for instance, to indicate a behavioral and psychological disturbance due to an organic condition different from dementia (i.e., *delirium*), to describe psychomotor activation due to different neuropsychiatric syndromes (e.g., anxiety or depression), or to indicate a BSPD lacking specific pharmacological treatment (e.g., wandering) (51).

An impulsive syndrome bears enormous risks for the patient and suffering for the caregiver. In Alzheimer's disease, this condition has been associated with extensive serotonergic deficits (52, 53), along with altered serotonergic modulation of dopaminergic neurotransmission (54). Given the differences in safety profile, selective serotonin reuptake inhibitors (SSRI) and antipsychotics are recommended, respectively, as first and second choice medications, in spite of the fact that evidence of symptomatic relief favors the formers (Table 2).

## Maniform Syndrome

The definition of maniform syndrome, independent of depression, is a novel contribution of the CHROME criteria. Its inclusion was considered necessary for several reasons. Also termed “elation” (15), mania-like symptoms are sometimes associated with impulsive/disinhibition symptoms (19, 30), but may also stand alone in cluster analyses of neuropsychiatric symptoms of dementia (20, 31). On the other hand, late-onset bipolar patients tend to have a milder illness in terms of manic severity and display a higher medical and neurological burden (55). In fact, mania may be observed in the elderly in the absence of depression or previous bipolar disorder, in association with cerebrovascular disease and other neurological conditions (56, 57). Moreover, those patients usually get worse with antidepressant medications (58). Hence, mania was defined as an individual syndrome, independent of impulsive syndrome, depression, and bipolar disorder, to better identify and treat these patients.

## Sleep Disturbance

Usually associated with aging, disruptions of normal circadian rhythms and sleep cycles are aggravated in neurodegenerative conditions due to melatonin decrease and other biological factors (59). However, sleep disturbance present enormous intra and interindividual variety along the course of dementia (45), possibly reflecting the influence of genetical, psychological, and environmental factors (60).

As a general strategy, the CHROME criteria recommend identifying a primary syndrome on which to build the choice of treatment for each clinical case (Table 1). That strategic approach is especially advisable for sleep disorder, which may be secondary to any other neuropsychiatric syndrome. In addition, the presence of other conditions (e.g., pain, periodic limb movements) should be carefully searched and specifically treated (60). In case of primary sleep alteration, melatonin (61), short-term use of benzodiazepines, and other medications are recommended, although the evidence of effect, for most medications, is very low (Table 2).

## Discussion

In historical terms, the dementia epidemic hit society unready. Reactions to dam its effects have in essence consisted in adapting previously existing solutions. Hospitals morphed into nursing homes, Kindergarten into day care facilities, charitable organizations into state-ruled social services. Occupational therapists set up reality orientation interventions based on previous rehabilitation programs for WWII victims (62). Drugs initially developed for psychiatric patients were incorporated for the treatment of highly disturbing, dementia-related symptoms.

At the dawn of the new millennium, the construct BPSD gave birth to a wide taxonomy of symptoms (16, 18). And with it came a symptom-based approach of psychotropic prescription. Research was scarce, controversy among specialist was great and helplessness of non-specialized physicians nagging. Meanwhile, societal demand on physicians for a “quick fix” was pressing. As the underlying causes of symptoms were less than clear, psychotropic treatments shifted to medications capable of decreasing the magnitude or frequency of a wide range of problematic symptoms. As expected, these drugs were frequently neuroleptics, benzodiazepines, and other drugs with sedative effects (65, 66).

The pernicious consequences of these prescriptions were soon identified and the advent of initiatives to promote deprescribing of antipsychotics and benzodiazepines was the next historical stage (4–6). Initiatives based on prescribing guidelines of specific drug types failed to yield satisfactory results, as for the non-specialized physician these guidelines were possibly either too complex, too limited in their symptomatic scope, or both (1, 2). The guideline by drug-type approach did not seem to solve the wider problems at stake (67–69). In a certain way, this narrow approach may even be part of the problem.

The two essential tools for medical success are: (1) accurate diagnostic tools and (2) efficacious treatments. The former and the latter are precisely what those guidelines do not provide, and the ultimate reason of their disappointing results. Knowing when not to prescribe an antipsychotic does not help the physician to make a sound treatment decision in a complex case.

To go beyond past narrow-focused experiences, the CHROME expert panel's objective was creating an all-encompassing tool for quality prescribing of psychotropics that included all the critical components non-specialized clinicians need: legal guideline, dementia-specific definition of chemical restraints, diagnostic criteria, recommended drugs, methodology to verify diagnostic and treatment quality by an independent observer, and good practice standards in pharmacy (21). For diagnostic guidance, the CHROME criteria were built on a comprehensive syndromic frame, which provides rationale for the pharmacological treatment, and which other scholars had already envisioned and investigated (19, 20, 70, 71).

Since its first publication in 2016 (21), CHROME criteria have been implemented in five nursing homes spread throughout Spain. Three homes have additionally passed an independent audit undertaken by the Spanish Alzheimer Society (CEAFA) that confirmed their diagnostic and treatment quality as CHROME criteria compliant. The fact that CHROME diagnostic criteria can be shared and agreed upon between medical doctors appears encouraging regarding their validity.

The large number of de-prescriptions that result when employing these criteria have given those physicians a tool to push back the pressure demanding medical solutions for problems outside the doctor's realm. Quality of care, environmental adequacy, and non-pharmacological approaches regain their value as soon as psychotropics are no option to compensate for shortcomings in these areas. In fact, homes that consistently apply CHROME criteria had to readjust certain organizational processes like bedtime routines, use of facilities, or how to liaise with families (63, 64).

Decades of drug- or symptom-based approaches have not attained satisfactory results. Our research indicates that CHROME diagnostic criteria and the associated drug recommendations are a significant step further (Table 2). However, diagnostic criteria and treatment recommendations lack a solid research base. Although CHROME syndrome definitions have been successfully applied, their neurobiological underpinnings and potential biomarkers remain to be reasonably elucidated. Explorations along these lines together with further characterization, including description of clinical diversity according to dementia etiology and severity should contribute to sharpen the picture of the processes underlying these clinical landscapes. Such efforts would in turn help to guide better pharmacological (and non-pharmacological) treatments and their research.

Overall, present quality of evidence to support the efficacy of psychotropic medications in dementia is weaker than the evidence that proves their risks (3, 27, 30, 50). This inconvenient fact justifies trial and error approaches in not uncommon cases where results are either unsatisfactory or absent. Although CHROME criteria have not managed to completely avoid some trial-and-error attitudes common in psychotropic prescription, its achievement has been limiting the potential number of options to try out as well as suggesting an order from the efficacy-safety perspective. The mentioned lack of sufficient evidence supporting the use of psychotropics for many problems is the reason why CHROME criteria recommend prescriptions only for the most severe cases.

Nonetheless, a certain laxity was built into these diagnostic criteria in favor of the physicians' clinical judgement, even permitting a “possible” diagnosis for patients for whom information was not sufficient or adequate (e.g., patients with advanced dementia, or without informants), there were doubts about the primary syndrome, or there were other medical, psychological, or social factors which could explain, totally or partially, the symptoms (Table 3). This deliberate flexibility regarding diagnostic certainty and cut off points affects prescribing and treatment response. It would be of interest researching how these criteria compare to earlier guidelines in the case of most dangerous drugs (e.g., neuroleptics).

**Table 3:** Frequency of CHROME syndromes in two independent samples^a^.

	**El Pino Study^b^**	**Albertia Study^c^**	**Pooled data**
	**(*n* = 214)**	**(*n* = 139)**	**(*n* = 353)**
**Depression**			
Possible	0.5	7.9	3.4
Certain	15.0	23.0	18.1
Total	15.4 (10.6–20.3)	30.9 (23.3–38.6)	21.5 (17.2–25.8)
**Anxiety**			
Possible	0.9	12.2	5.4
Certain	31.3	25.2	28.9
Total	32.2 (26.0–38.5)	37.4 (29.4–45.5)	34.3 (29.3–39.2)
**Psychotic syndrome**			
Possible	0.5	9.4	4.0
Certain	6.5	10.8	8.2
Total	7.0 (3.6–10.4)	20.1 (13.5–26.8)	12.2 (8.8–15.6)
**Impulsive syndrome**			
Possible	0.0	1.4	0.6
Certain	7.9	10.1	8.8
Total	7.9 (4.3–11.6)	11.5 (6.2–16.8)	9.3 (6.3–12.4)
**Maniform syndrome**			
Possible	0.0	0.7	0.3
Certain	0.9	0.7	0.8
Total	0.9 (0.0–2.2)	1.4 (0.0–3.4)	1.1 (0.0–2.2)
**Sleep disturbance**			
Possible	0.0	11.5	4.5
Certain	12.2	21.6	15.9
Total	12.2 (7.8–16.6)	33.1 (25.3–40.9)	20.4 (16.2–24.6)

a *The frequency (95% confidence interval) of possible, certain, and possible or certain syndrome is given*;

b *observational, prospective study in a single nursing home (mean age [SD] 81.3 [10.7], 61.6% of the residents were female, 77.4% had dementia) (63)*;

c *observational, prospective study in two nursing homes (mean age [SD] 88.1 [5.6], 80.6% of the residents were female, 84.8% had dementia (64)*.

CHROME criteria have proven safe, to increase quality of life and to lower usual psychotropic prescribing between half and two thirds (63, 64). Additionally, applying CHROME criteria make chemical restraint prescribing virtually impossible from the get-go or when used for medication review. Hence, usefulness of CHROME criteria seems established and specific lines for future research regarding diagnostic reliability and treatment options are also envisaged. It remains to be seen if clinicians and researchers are able or willing to try out the paradigm review implicit in them.

## Data Availability Statement

The data analyzed in this study is subject to the following licenses/restrictions: The data set is available, from the PI, upon request. Requests to access these datasets should be directed to javier@mariawolff.es.

## Author Contributions

JO conceived the objective and structure of the perspective paper, drafted and wrote most parts of the manuscript, supervised, and performed all statistical analyses. RM wrote significant parts of the manuscript, performed data analysis, and incorporated contributions of JL-A, LA-O, JO, and LP. JL-A and LA-O wrote significant parts regarding diagnostic syndrome criteria and treatment. LP provided data, ensured data integrity, and interpreted data. All authors contributed to manuscript revision, read and approved the submitted version.

## Conflict of Interest

The authors declare that the research was conducted in the absence of any commercial or financial relationships that could be construed as a potential conflict of interest.
